# Residual force depression in single sarcomeres is abolished by MgADP-induced activation

**DOI:** 10.1038/srep10555

**Published:** 2015-06-03

**Authors:** Neal Trecarten, Fabio C. Minozzo, Felipe S. Leite, Dilson E. Rassier

**Affiliations:** 1 Department of Kinesiology and Physical Education, McGill University; 2 Departments of Physics and Physiology, McGill University

## Abstract

The mechanisms behind the shortening-induced force depression commonly observed in skeletal muscles remain unclear, but have been associated with sarcomere length non-uniformity and/or crossbridge inhibition. The purpose of this study was twofold: (i) to evaluate if force depression is present in isolated single sarcomeres, a preparation that eliminates sarcomere length non-uniformities and (ii) to evaluate if force depression is inhibited when single sarcomeres are activated with MgADP, which biases crossbridges into a strongly-bound state. Single sarcomeres (n **=** 16) were isolated from rabbit psoas myofibrils using two micro-needles (one compliant, one rigid), piercing the sarcomere externally adjacent to the Z-lines. The sarcomeres were contracted isometrically and subsequently shortened, in both Ca^2+^- and MgADP-activating solutions. Shortening in Ca^2+^-activated samples resulted in a 27.44 **±** 9.04% force depression when compared to isometric contractions produced at similar final sarcomere lengths (P **<** 0.001). There was no force depression in MgADP-activated sarcomeres (force depression **=** −1.79 **±** 9.69%, P **=**  0.435). These results suggest that force depression is a sarcomeric property, and that is associated with an inhibition of myosin-actin interactions.

When a shortening is imposed to an activated skeletal muscle, the steady-state force generated after shortening is lower than that produced during isometric contraction at a similar sarcomere length (SL)^1^,^2^,^3^,^4^. This phenomenon is inconsistent with predictions based on the isometric force-length relationship^5^, and is commonly referred to as force depression. Force depression has been observed for more than half a century in different muscle preparations, from whole muscles^6^,^7^ to single fibres^2^,^8^ and myofibrils^9^,^10^. Its mechanisms, however, remain unclear.

A non-uniform distribution of the lengths of sarcomeres after shortening has been suggested to explain force depression^11^,^12^, as follows: when a muscle is activated and then shortened, the sarcomeres would shorten to different lengths^11^. A first group of sarcomeres would shorten by a small amount, while another group of sarcomeres would shorten to the ascending limb of the force-length relationship until the force produced by the first group is matched. As a result, the force generated by these two groups of sarcomeres would be lower than that predicted by the average sarcomere length of both populations together^12^. Although generally accepted, such a mechanism has been challenged by studies demonstrating the existence of force depression in muscle fibres in which the sarcomere lengths were controlled and kept stable both during and after shortening^2^,^13^, suggesting a mechanism intrinsic to the sarcomere.

Marechal and Plaghki^14^ had suggested many years ago that a shortening-induced strain in the actin filaments could cause an inhibition of the crossbridge binding sites on actin’s newly formed overlap zone during shortening, decreasing the probability of crossbridge formation, and consequently attenuating force production. A recent study from our laboratory suggests that this mechanism may work at the myofibril level; we observed that force depression was present in myofibrils without a concomitant increase in sarcomere length non-uniformity^9^. Most tellingly, force depression was partially inhibited by inducing strong binding of crossbridges and actin^9^, decreasing the crossbridge inhibition that may have been caused by the strain of actin filaments.

In order to clearly elucidate the mechanisms of force depression, it is necessary to isolate the contribution of *sarcomeric* properties from *between*-sarcomere properties. According to the sarcomere length non-uniformity theory, at least two sarcomeres in series are necessary to produce residual force depression after shortening. Therefore, the goals of this study were: (i) to examine if force depression was present in single sarcomeres, and (ii) to examine if force depression could be prevented by increasing crossbridge binding during/after shortening. In order to reach these goals, we used a single sarcomere preparation developed in our laboratory^15^, that was activated either with Ca^2+^ or MgADP – the latter biasing crossbridges into a strong binding state with actin^9^.

## Results

**Figure 1:**
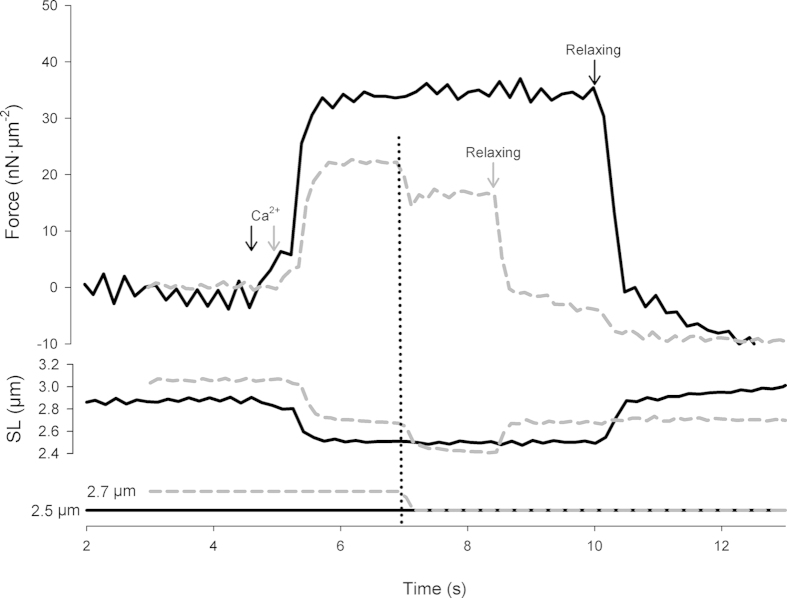
Sample records from a typical experiment displaying superimposed force, SL, and ramp traces of a Ca^2+^-activated sarcomere. The solid black line represents the isometric contraction. The dashed grey line represents the contraction in which a shortening was imposed. The vertical dotted line represents the time (~7 s) at which the imposed shortening occurred in the shortening condition. The black and grey arrows indicate the time at which the activating and relaxing solutions were flushed into the experimental chamber, in the isometric and shortening contraction, respectively.

Figure 1 shows a typical contraction produced by a sarcomere during Ca^2+^ activation, with the corresponding SL and ramp traces. After ~5 s, Ca^2+^-activating solution was flushed into the experimental chamber and the sarcomere was activated. The force stabilized after ~1 s. In the shortening contraction, the imposed shortening occurred at ~7 s, resulting in a similar final SL in the isometric contraction. Relaxing solution was flushed once a steady-state force was re-established after the imposed shortening, at ~8.5 s. In the isometric contraction, relaxing solution was administered after ~5 s at a maximum steady-state force, at ~10.5 s. The black and grey arrows indicate the timing of solution administration in the isometric and shortening contraction, respectively.

**Figure 2:**
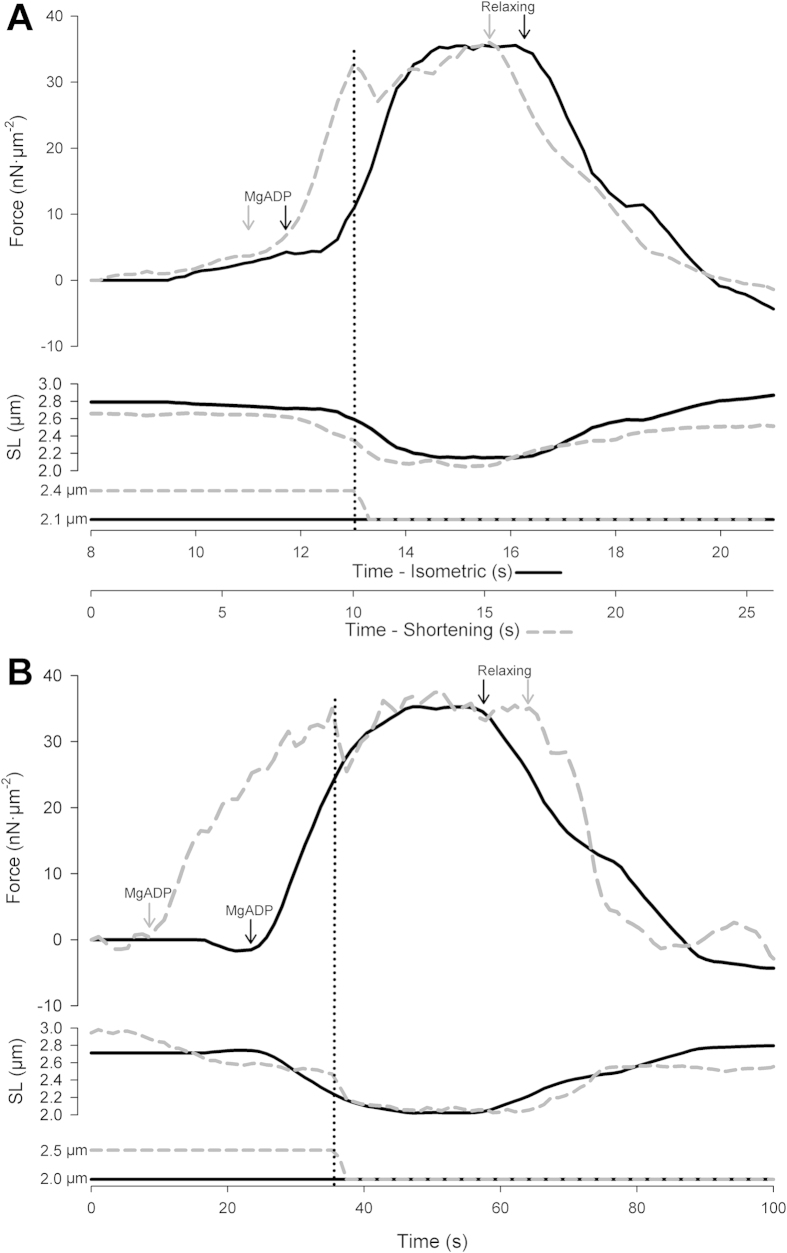
**A - Sample records from a typical experiment displaying superimposed force, SL, and ramp traces of a MgADP-activated sarcomere.** The solid black line represents the isometric contraction. The dashed grey line represents the contraction in which a shortening was imposed. The vertical dotted line represents the time at which the imposed shortening occurred (~10 s) in the shortening condition. The black and grey arrows indicate the time at which the activating and relaxing solutions were flushed into the experimental chamber, in the isometric and shortening contraction, respectively. **B - Sample records from a typical experiment displaying superimposed force, SL, and ramp traces of a sarcomere that was activated with MgADP for a longer period of time.** The solid black line represents the isometric contraction. The dashed grey line represents the contraction in which a shortening was imposed. The vertical dotted line represents the time at which the imposed shortening occurred (~10 s) in the shortening condition. The black and grey arrows indicate the time at which the activating and relaxing solutions were flushed into the experimental chamber, in the isometric and shortening contraction, respectively.

Figure 2 shows two superimposed graphs of force, length and ramp traces in MgADP-activated sarcomere. The force plateau of the isometric was time-matched with the force plateau upon force redevelopment in the shortening contraction in order to compare force at the same final SL. In Fig. 2A, in the isometric contraction, activation was induced at 12 s, and in the shortening contraction, activation was induced at 7 s – as shown by the black and grey arrows, respectively. The imposed shortening occurred at ~10 s after the force has maximized, and we compared the steady-state force generated in both corresponding contractions at ~14.5, which are approximately at the same final SL. Relaxing solution was flushed at 16.5 s, as indicated by the arrows, and the force stabilized to zero. Note that that the shortening is imposed slightly before a steady-state force is achieved. It has been shown that a steady-state force is not required prior to active shortening to produce force depression^8^,^12^. Due to the longer duration needed to attain maximal force using MgADP^9^, the duration of experiments was increased prior to shortening. This procedure does not affect the comparisons made between contractions. The traces in Fig. 2B shows another experiment in which MgADP activation was held for 35 s prior to the imposed shortening, and the force depression was similar to the experiment depicted in Fig. 2A. Note that the force was still increasing over time, although it was slowly reaching a plateau towards the end of the contraction.

**Figure 3:**
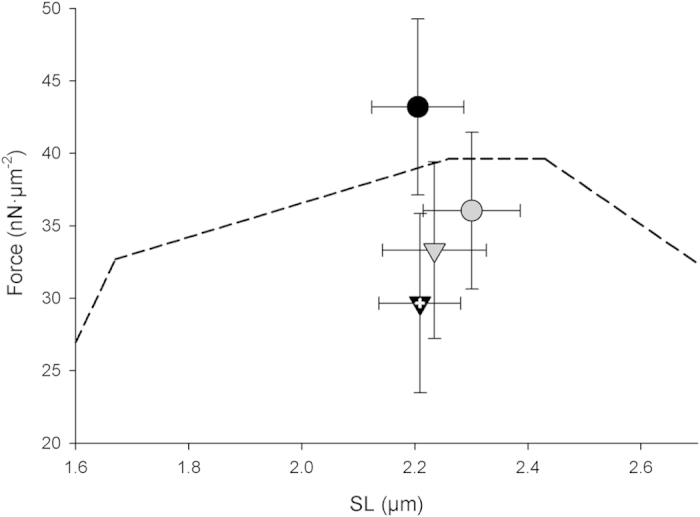
Mean values of the SL-force relation for each experimental condition. Black circle represents Ca^2+^-activated, isometric contractions. Black triangle with white cross represents Ca^2+^-activated, imposed shortening contractions. Grey circle represents MgADP-activated, isometric contractions. Grey triangle represents MgADP-activated, imposed shortening contractions. White cross represents statistically significant force depression relative to isometric counterpart (P 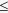 0.05). The dashed line represents the force-length relationship based on the mean isometric force generated by all sarcomeres (both Ca^2+^- and MgADP-activated samples).

The results from Figs. 1,2 were confirmed statistically as shown in Fig. 3, which illustrates the mean forces produced in all conditions. Post hoc analysis using the Holm-Sidak method indicated that the only significant difference in force existed between Ca^2+^-activated isometric (43.21 ± 6.08 nN/μm^2^) and shortening contractions (29.66 ± 6.19 nN/μm^2^) (*t*(15) = 3.939, *p* < 0.001). The final SL and the amount of shortening were not different between Ca^2+^- and MgADP-activation. The imposed shortening in Ca^2+^-activated samples resulted in a force depression of 27.44 ± 9.04% whreas force depression was virtually eliminated when the sarcomere was activated by MgADP.

## Discussion

The current study is the first to show that the shortening-induced force depression commonly observed in skeletal muscles is present at the single sarcomere level with Ca^2+^ activation. Furthermore, this study shows that force depression is abolished when samples are activated with MgADP, which biases crossbridges into a strongly-bound state. These findings suggest that force depression is caused partly by crossbridge binding site inhibition. Such a mechanism would be consistent with an actin distortion in the newly formed overlapped zone during shortening^9^,^14^.

When single sarcomeres were activated with Ca^2+^ and subsequently shortened, force was noticeably depressed in comparison to the isometric contraction at the corresponding final SL. One suggested mechanism for force depression is sarcomere length non-uniformities^11^,^12^ – a result of strength differences between sarcomeres in series. The heterogeneity in sarcomere length shortening results in some sarcomeres being longer, lying on the descending limb of the force-length relationship, and sarcomeres on the ascending limb matching the force produced by the other group of sarcomeres. The actual force produced is thus less than that predicted during isometric contractions. While the observation of force depression in Ca^2+^-activated samples in our study is consistent with previous studies using muscle fibres and myofibrils^2^,^7^,^9^, this study is the first to assess the magnitude of force depression when removing the contribution of non-uniformity between sarcomeres.

Previously, we observed that force depression was greatly reduced when myofibrils were activated with MgADP^9^. In the present study, we showed a full abolishment of force depression when single sarcomeres were activated in MgADP solution. The difference may reside in the fact that in myofibril preparations, some degree of sarcomere length non-uniformities may contribute to the overall force depression.

The absence of force depression in MgADP-activated samples supports a mechanism of force depression, intrinsic to the sarcomere, that has been suggested many years ago; a shortening-induced inhibition of myosin-actin attachment in the newly formed overlap zone during shortening^14^. Such an inhibition may be caused by a stress-deformation of actin, hindering its myosin-binding sites. Activation via MgADP happens when ADP enters the myosin nucleotide-binding pocket, inducing some crossbridges to transit into a strongly-bound state^16^. The strong binding of myosin-actin in turn may introduce conformational changes in the tropomyosin filaments, cooperatively exposing adjacent myosin-binding sites on actin, ultimately leading to further force-generating crossbridge attachments^17^,^18^. Furthermore, the strong binding from MgADP-activation has been suggested to compress the myofilament lattice^19^, pulling actin and myosin closer together.

It is tempting to speculate that MgADP activation allows the sarcomere to counteract and overcome the shortening-induced force depression by improving the alignment and resultant probability of actomyosin formation. MgADP induces strong binding between myosin crossbridges and actin in a cooperative fashion. Studies performed in solution with reconstituted filaments show that strong biding of myosin crossbridges to actin filament induces the binding of adjacent crossbridges^20^,^21^. Such strongly-bound crossbridges may cause conformational changes of the thin filaments that may in fact enhance P_i_ release or other steps during isomerization of the actin-myosin–ADP–P_i_ complex. Another effect of strong binding between crossbridges and actin is the compression of the myofilament lattice space upon muscle activation^19^, approximating thick and thin filaments. A decreased lattice space will likely increase the probability of crossbridge attachment to actin, which could counteract some of the shortening induced depression effects on force. Finally, the rigidity of the actin filament is reduced upon strong binding of myosin crossbridges^22^, which would release the stress caused by activation and shortening to the thin filaments. Altogether, these observations as well as our results indicate that the main difference between sarcomeres activated with Ca^2+^ or MgADP is the myosin–actin binding probability. When myofibrils are activated and shortened with continuous flow of MgADP, the strong binding of crossbridges will be enhanced, increasing myosin cooperativity while decreasing the lattice spacing and the rigidity of the actin filaments. The newly formed overlap zone after shortening will be better aligned for myosin binding to actin.

In summary, this study was the first to provide direct support for a purely sarcomeric mechanism of force depression. While between-sarcomere mechanisms cannot be discounted in myofibril preparations, the observation that MgADP-activation abolishes force depression suggests that force depression is caused by an inhibition of myosin-actin interactions in the newly formed overlap zone during shortening.

## Methods

### Muscle Preparation and Single Sarcomere Isolation

Sections of rabbit psoas muscles (3-4 cm) were dissected and subject to a standard permeabilization process^23^. For four hours, the muscles were bathed in rigor solution (pH = 7.0), after which the samples were placed in a rigor:glycerol (50:50) solution overnight. The samples were then transferred to a new rigor:glycerol solution with an additional mixture of protease inhibitors (Roche Diagnostics, USA). The muscles were frozen (−20 °C) for at least seven days. The protocol was approved by the McGill University Animal Care Committee and complied with the guidelines of the Canadian Council on Animal Care.

Small sections of a muscle sample were cut and placed in a 2 mL Eppendorf tube containing fresh rigor solution. The sample was thawed for one hour in the fridge (4 °C), and then transferred to approximately 5 mL of rigor solution in a 10 mL test tube. This sample was then homogenized using the following procedure: twice for 5 s at 18,000 rpm, twice for 5 s at 26,000 rpm and once for 3 s at 34,000 rpm. The homogenate, containing single myofibrils, was pipetted onto a glass cover-slip (thickness: 0.15 mm), which was vacuum grease-sealed to an experimental chamber positioned on the stage of an inverted microscope (NIKON Eclipse TE 2000U). Surrounding the chamber, a circulating cooling solution controlled the temperature at 10 °C. After 10 minutes, the homogenate was replaced with a relaxing solution (pH 7.0), which was subsequently used to fill the chamber, minimizing the appearance of floating debris. A myofibril was chosen for mechanical experimentation based on its striation pattern appearance, upon which a single sarcomere could be selected for isolation.

### Solutions

The rigor solution (pH 7.0) was composed of (in mM): 50 Tris, 100 KCl, 2 MgCl_2_ and 1 EGTA. The relaxing solution (pH 7.0) was composed of (in mM): 0.01372 CaCl2, 7.2 EGTA, 20.27 Imidazole, 5.41 MgCl_2_, 68.678 KCl, 6.1 ATP and 18.97 creatine phosphate. Two activating solutions were used in this investigation. The Ca^2+^-activating solution (pH 7.0) was composed of (in mM): 7 CaCl_2_, 7.2 EGTA, 20.27 Imidazole, 5.41 MgCl_2_, 52.31 KCl, 6.169 ATP and 18.97 creatine phosphate. The MgADP-activating solution (20 mM) (pH 7.0) was composed of (in mM): 2 MgCl_2_, 20 MOPS, 4 EGTA, 4 ATP and 20 ADP.

### Micro-needle production and calibration

A vertical pipette puller (KOPF 720, David Kopf Instruments) was used to make two glass micro-needles. Calibration of the micro-needles was done using a cross-bending method using a pair of micro-fabricated cantilevers of known stiffness (51.8 and 52.6 nN/μm)^24^. For each experiment, a thin, compliant micro-needle (stiffness varied between 30 and 110 nN/μm) and a thick, rigid micro-needle were used.

### Mechanical isolation, visualization, and force measurement of single sarcomeres

Using the two pre-calibrated micro-needles controlled by micromanipulators (Narishige NT-88-V3, Tokyo, Japan), single sarcomeres were pierced externally adjacent to Z-lines. The sarcomeres were raised from the glass coverslip by 0.5-1.0 μm. Under high magnification provided by an oil immersion phase-contrast lens (Nikon plan-fluor, X100, numerical aperture 1.30), the images of the single sarcomeres were further magnified X1.5 by an internal microscope function. A video was taken throughout sarcomere isolation and mechanical experimentation.

After the mechanical isolation of the sarcomere, a contraction was initiated by a computer-controlled release of the activating solution through a double-barreled pipette connected to a multichannel perfusion system (VC-6M, Harvard Apparatus)^15^,^25^. To avoid contamination within the perfusion system, each side of the double-barrel was equipped to flush its own, independent activating solution contacting Ca^2+^ or MgADP, as well as relaxing solution.

The contrast between the micro-needles produces a pattern of light intensity peaks that allow for tracking of their centroids frame-by-frame using a particle-tracker algorithm. The force produced during activation of the single sarcomere was obtained by measuring the displacement of the micro-needles, as previously described^15^.

### Experimental Protocol

Single sarcomeres (n = 16) were first activated isometrically and relaxed after force reached a steady-state for several seconds. Activation was induced in separate contractions using either Ca^2+^ or MgADP solutions. Subsequently, the same sarcomere was passively stretched to a longer sarcomere length and activated. After force reached a steady-state (Ca^2+^ activation) or a quasi-steady-state (MgADP activation), a computer-controlled shortening with an amplitude of 0.40 ± 0.03 μm at a speed of 203.14 ± 14.45 nm.s^−1^ was imposed by the rigid micro-needle. In order to clearly identify force depression, the potential difference in forces was always investigated at the same final SL’s. Relaxing solution was flushed into the experimental bath in order to stop the contractions. It is known that, during MgADP activation, the rate of force development is significantly slower than that produced during Ca^2+^ activation^9^, and it may take up to a few minutes. Therefore, shortening was not always imposed during steady-state force in MgADP-activated sarcomeres, and in some cases force was still rising. However, it has been shown that force depression is present when shortening is imposed in the force rising phase, at levels that are qualitatively similar to those observe when shortening is imposed during the plateau of the force response in fully activated muscle fibres^8^,^9^,^10^,^11^,^12^.

**Figure 4:**
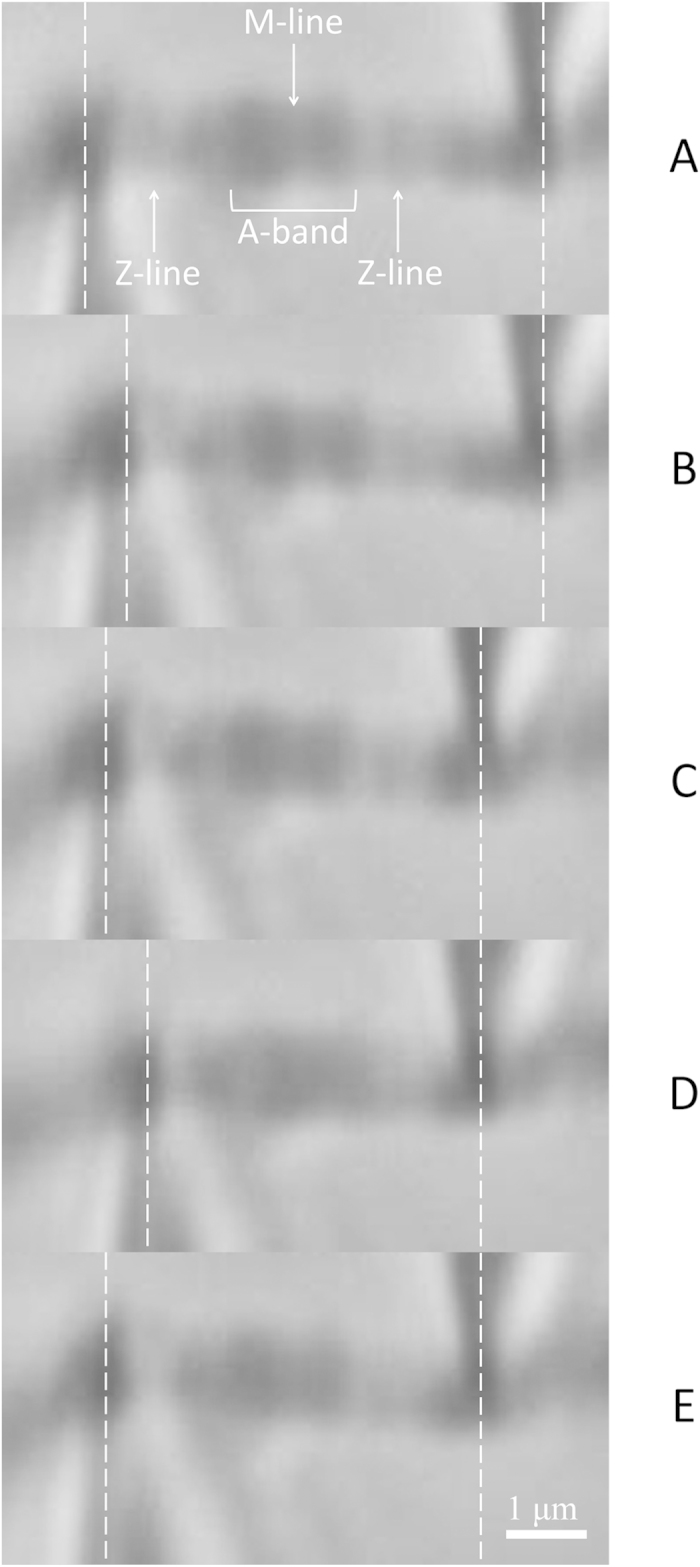
Needle movement through each phase of an MgADP-activated, shortening contraction. The white dashed lines represent the midpoint of the needle at each phase of the contraction. The right needle (which imposes the computer-controlled shortening) is ~10x stiffer than the left needle, which moves in response to any sarcomere length changes (activation, relaxation, and imposed shortening). **A**) Baseline – represents the initial position of both needles; **B**) Activation – the sarcomere is activated and contracts; **C**) Shortening – a shortening is imposed by the thick needle, decreasing the sarcomere length while moving the thin needle in the process; **D**) Re-development – the sarcomere contracts at this new, shorter sarcomere length as crossbridges begin to reattach; **E**) Relaxation – the relaxing solution is flushed into the preparation.

The order of different activations (Ca^2+^ and MgADP) and contraction types (isometric and shortening) was randomized to account for confounding effects. Figure 4 shows the needle movement through each phase of a typical MgADP-activated, shortening contraction.

### Data Analysis

The amount of force depression was measured by calculating the difference between the isometric force and the force produced after the imposed shortening in each sarcomere. The values taken for the calculation were averaged over 1 second in both contractions.

The results are presented as means ± standard error (SEM). Since the data were not normally distributed, a two-way ANOVA for repeated measures on ranks (n = 16) was used to identify potential differences in force and final SL between the four experimental conditions between groups. The Holm-Sidak post-hoc test was used to locate these differences when they were present. A level of significance (P) was set at 0.05. Since there is a relationship between the amount of shortening and active force depression, a student’s t-test (n = 12) was used to compare the amount of shortening between the two activating solutions.

## Additional Information

**How to cite this article**: Trecarten, N. *et al.* Residual force depression in single sarcomeres is abolished by MgADP-induced activation. *Sci. Rep.* **5**, 10555; doi: 10.1038/srep10555 (2015).

## References

[b1] Edman K. A. Mechanical deactivation induced by active shortening in isolated muscle fibres of the frog. J. Physiol. 246, 255–275 (1975). 1079534 10.1113/jphysiol.1975.sp010889 PMC1309413

[b2] Granzier H. L. & Pollack G. H. Effect of active pre-shortening on isometric and isotonic performance of single frog muscle fibres. J. Physiol. 415, 299–327 (1989). 2640463 10.1113/jphysiol.1989.sp017723 PMC1189178

[b3] Morgan D. L., Claflin D. R. & Julian F. J. Tension in frog single muscle fibers while shortening actively and passively at velocities near Vu. Biophys. J. 57, 1001–1007 (1990). 2340339 10.1016/S0006-3495(90)82619-9 PMC1280806

[b4] Herzog W., Leonard T. R. & Wu J. Z. Force depression following skeletal muscle shortening is long lasting. J. Biomech. 31, 1163–1168 (1998). 9882049 10.1016/s0021-9290(98)00126-2

[b5] Gordon A. M., Huxley A. F. & Julian F. J. The variation in isometric tension with sarcomere length in vertebrate muscle fibres. J. Physiol. 184, 170–192 (1966). 5921536 10.1113/jphysiol.1966.sp007909 PMC1357553

[b6] Abbott B. C. & Aubert X. The force exerted by active striated muscle during and after change of length. J. Physiol. 117, 77–86 (1952). 14946730 PMC1392571

[b7] Herzog W. & Leonard T. R. Depression of cat soleus-forces following isokinetic shortening. J. Biomech. 30, 865–872 (1997). 9302608 10.1016/s0021-9290(97)00046-8

[b8] Edman K. A., Caputo C. & Lou F. Depression of tetanic force induced by loaded shortening of frog muscle fibres. J. Physiol. 466, 535–552 (1993). 8410705 PMC1175491

[b9] Pun C., Syed A. & Rassier D. E. History-dependent properties of skeletal muscle myofibrils contracting along the ascending limb of the force-length relationship. Proc. R. Soc B 277, 475–484, 10.1098/rspb.2009.1579 (2010). PMC2842654 19846455

[b10] Joumaa V. & Herzog W. Force depression in single myofibrils. J. Appl. Physiol. 108, 356–362, 10.1152/japplphysiol.01108.2009 (2010). 20007852

[b11] Julian F. & Morgan D. The effect on tension of non-uniform distribution of length changes applied to frog muscle fibres. J. Physiol 293, 379–392 (1979). 315465 10.1113/jphysiol.1979.sp012895 PMC1280719

[b12] Morgan D., Whitehead N., Wise A., Gregory J. & Proske U. Tension changes in the cat soleus muscle following slow stretch or shortening of the contracting muscle. J. Physiol. 522, 503–513 (2000). 10713973 10.1111/j.1469-7793.2000.t01-2-00503.x PMC2269772

[b13] Sugi H. & Tsuchiya T. Stiffness changes during enhancement and deficit of isometric force by slow length changes in frog skeletal muscle fibres. J. Physiol. 407, 215–229 (1988). 3256616 10.1113/jphysiol.1988.sp017411 PMC1191199

[b14] Marechal G. & Plaghki L. The deficit of the isometric tetanic tension redeveloped after a release of frog muscle at a constant velocity. J. Gen. Physiol. 73, 453–467 (1979). 312915 10.1085/jgp.73.4.453 PMC2215170

[b15] Minozzo F. C., Baroni B. M., Correa J. A., Vaz M. A. & Rassier D. E. Force produced after stretch in sarcomeres and half-sarcomeres isolated from skeletal muscles. *Sci*. Rep. 3, 2320, 10.1038/srep02320 (2013). PMC3728588 23900500

[b16] Kintses B. *et al.* Reversible movement of switch 1 loop of myosin determines actin interaction. EMBO J. 26, 265–274 (2007). 17213877 10.1038/sj.emboj.7601482 PMC1782383

[b17] Shimizu H., Fujita T. & Ishiwata S. i. Regulation of tension development by MgADP and Pi without Ca^2+^. Role in spontaneous tension oscillation of skeletal muscle. Biophys. J. 61, 1087–1098 (1992). 1600074 10.1016/S0006-3495(92)81918-5 PMC1260373

[b18] Gordon A. M., Homsher E. & Regnier M. Regulation of contraction in striated muscle. Physiol Rev. 80, 853–924 (2000). 10747208 10.1152/physrev.2000.80.2.853

[b19] Brenner B. & Yu L. C. Equatorial x-ray diffraction from single skinned rabbit psoas fibers at various degrees of activation. Changes in intensities and lattice spacing. Biophys. J. 48, 829–834 (1985). 4074840 10.1016/S0006-3495(85)83841-8 PMC1329408

[b20] Bremel R. D. & Weber A. Cooperation within actin filament in vertebrate skeletal muscle. Nat. New Biol. 238, 97–101 (1972). 4261616 10.1038/newbio238097a0

[b21] Greene L. E. & Eisenberg E. Cooperative binding of myosin subfragment-1 to the actin-troponin-tropomyosin complex. Proc. Natl. Acad. Sci. USA 77, 2616–2620 (1980). 6930656 10.1073/pnas.77.5.2616 PMC349453

[b22] Yanagida T., Nakase M., Nishiyama K. & Oosawa F. Direct observation of motion of single F-actin filaments in the presence of myosin. Nature 307, 58–60 (1984). 6537825 10.1038/307058a0

[b23] Minozzo F. C., Hilbert L. & Rassier D. E. Pre-power-stroke cross-bridges contribute to force transients during imposed shortening in isolated muscle fibers. PloS ONE 7, e29356, 10.1371/journal.pone.0029356 (2012). 22242168 PMC3252314

[b24] Pavlov I., Novinger R. & Rassier D. E. The mechanical behavior of individual sarcomeres of myofibrils isolated from rabbit psoas muscle. *Am. J. Physiol* - Cell Ph. 297, C1211–C1219 (2009). 10.1152/ajpcell.00233.2009 19710362

[b25] Rassier D. E. & Pavlov I. Force produced by isolated sarcomeres and half-sarcomeres after an imposed stretch. Am. J. Physiol - Cell Ph. 302, C240–248, 10.1152/ajpcell.00208.2011 (2012). 21998143

